# APC^CDH1^ Targets MgcRacGAP for Destruction in the Late M Phase

**DOI:** 10.1371/journal.pone.0063001

**Published:** 2013-05-16

**Authors:** Koutarou Nishimura, Toshihiko Oki, Jiro Kitaura, Shinji Kuninaka, Hideyuki Saya, Asako Sakaue-Sawano, Atsushi Miyawaki, Toshio Kitamura

**Affiliations:** 1 Division of Cellular Therapy, The Institute of Medical Science, The University of Tokyo, Minato-ku, Tokyo, Japan; 2 Division of Stem Cell Signaling, The Institute of Medical Science, The University of Tokyo, Minato-ku, Tokyo, Japan; 3 Division of Gene Regulation, Institute for Advanced Medical Research, Keio University, School of Medicine, Shinjuku-ku, Tokyo, Japan; 4 CREST, Japan Science and Technology Agency, Chiyoda-ku, Tokyo, Japan; 5 Laboratory for Cell Function and Dynamics, Advanced Technology Development Group, Brain Science Institute, RIKEN, Wako-city, Saitama, Japan; 6 Life Function and Dynamics, ERATO, JST, Wako-city, Saitama, Japan; Institute of Medical Science, University of Tokyo, Japan

## Abstract

**Background:**

Male germ cell RacGTPase activating protein (MgcRacGAP) is an important regulator of the Rho family GTPases — RhoA, Rac1, and Cdc42 — and is indispensable in cytokinesis and cell cycle progression. Inactivation of RhoA by phosphorylated MgcRacGAP is an essential step in cytokinesis. MgcRacGAP is also involved in G1-S transition and nuclear transport of signal transducer and activator of transcription 3/5 (STAT3/5). Expression of MgcRacGAP is strictly controlled in a cell cycle-dependent manner. However, the underlying mechanisms have not been elucidated.

**Methodology/Principal Findings:**

Using MgcRacGAP deletion mutants and the fusion proteins of full-length or partial fragments of MgcRacGAP to mVenus fluorescent protein, we demonstrated that MgcRacGAP is degraded by the ubiquitin-proteasome pathway in the late M to G1 phase via APC^CDH1^. We also identified the critical region for destruction located in the C-terminus of MgcRacGAP, AA537–570, which is necessary and sufficient for CDH1-mediated MgcRacGAP destruction. In addition, we identified a PEST domain-like structure with charged residues in MgcRacGAP and implicate it in effective ubiquitination of MgcRacGAP.

**Conclusions/Significance:**

Our findings not only reveal a novel mechanism for controlling the expression level of MgcRacGAP but also identify a new target of APC^CDH1^. Moreover our results identify a C-terminal region AA537–570 of MgcRacGAP as its degron.

## Introduction

Male germ cell Rac GTPase activating protein (MgcRacGAP) is one of the most important regulators of the Rho family of GTPases — RhoA, Rac1, and Cdc42 [1], [2]. Rho family proteins are involved in many cellular functions — including cell morphology, migration, gene expression, apoptosis, and proliferation — via regulation of the cytoskeleton [3]. They also play important roles in cytokinesis and G1-S transition. To maintain normal cell cycle progression, it is essential to control their activities [4].

In terms of cell cycle control, two GTPase-activating proteins (GAPs), MgcRacGAP and p190RhoGAP, and two guanine nucleotide exchange factors (GEFs), Ect2 and GEF-H1, co-ordinately regulate Rho family GTPases [5]–[8]. As we and others have reported, Ect2 activates RhoA required for the initiation of cytokinesis, and MgcRacGAP localized in the midbody inactivates RhoA by its Rho-GAP activity induced by Aurora B kinase via phosphorylation at S387 at the end of mitosis [9]–[11]. The latter step is critical for completion of cytokinesis. MgcRacGAP depletion results in impairment of cell division *in vitro* [12]–[15] and *in vivo* [16], [17]. MgcRacGAP is also involved in inactivating another Rho family protein, Cdc42 in metaphase [18], and in localizing molecules including RhoA [19], CENP-A [20], and STAT3/5 [21]–[25]. Although the expression [26] and activity [27]–[29] of MgcRacGAP are strictly controlled during the cell cycle, the precise mechanisms have not been elucidated so far.

Degradation of proteins by the ubiquitin-proteasome pathway is a major means of regulating the cell cycle. Anaphase-promoting complex/cyclosome (APC/C) and Skp/Cullin/F-box (SCF) complexes have an E3 ligase activity both to mediate ubiquitination and to initiate proteasomal destruction of their target proteins cell cycle-dependently [30]–[33]. Several proteins involved in mitosis are the targets of APC/C, including Cyclin A/B, Securin, Geminin [33], Aurora A/B [34], [35], Ect2 [36] and p190RhoGAP [37]. APC/C is activated by binding either of the co-activators Cdc20 or Cdh1. APC^Cdc20^ becomes functional in the metaphase-anaphase transition and APC^cdh1^ is activated in late mitosis to the G1 phase [32], [33].

In the present study, we demonstrated that MgcRacGAP is degraded by the ubiquitin-proteasome pathway in the late M to G1 phase and that MgcRacGAP is a novel target of APC^Cdh1^. We also identify a critical region for destruction located in the C terminus of MgcRacGAP, AA537–570, which is necessary and sufficient for CDH1-mediated MgcRacGAP destruction. We were not able to identify the lysine residues or any functional motif for ubiquitination in this region. However, a PEST domain-like structure with charged residues was identified, which may be responsible for effective ubiquitination of MgcRacGAP. Thus, in this report, we have identified MgcRacGAP as a target of APC^Cdh1^, indicating a novel mechanism controlling the expression level of MgcRacGAP through cell cycle progression.

## Materials and Methods

### Cell line and cell culture

293T and NIH3T3 cells were cultured in D-MEM (Wako, Osaka, Japan) supplemented with antibiotics and 10% fetal calf serum (DMEM/10% FCS). 293T and NIH3T3 cells were obtained from ATCC. Mouse embryonic fibroblast (MEF) cells were cultured in DMEM/10% FCS on 100-mm gelatin-coated dishes. Immortalized MEFs derived from WT and *Cdh1* GT/GT mice were produced as described previously [37].

### Plasmids

Flag-tagged deletion mutants (ΔMyo, ΔInt, ΔCys, ΔGAP, Δ410–632, Δ463–632, Δ537–632, Δ611–632, Δ628–632, ΔGAP+CT, Δ537–570, and Δ537–570-ΔDbox) were generated using PCR. Flag-tagged or mVenus-tagged or mCherry-tagged wild-type and mutants of MgcRacGAP were cloned into *Eco*RI and *Not*I sites of the retrovirus vector pMXs-IRES-EGFP (pMXs-IG) or pMXs-IRES-Puro^r^ (pMXs-IP) [13] or the mammalian expression vector pME18S. Full-length or partial fragments of the MgcRacGAP C-terminal region were generated using PCR and cloned into *Eco*RI and *Not*I sites of a retrovirus vector pMXs-mVenusNLS-IRES-blasticidin^r^ to make fusion proteins between mVenus containing SV40-NLS (PKKKRKV) (mVenus-NLS) and full-length or partial fragments of the C-terminal portion of MgcRacGAP (CT). PCR was carried out using Phusion DNA polymerase (Thermo Fisher Scientific, Waltham, USA). pCSII-MCS-based vectors for AmCyan-hGeminin (1/110) and mCherry-hCdt1 (30/120) were previously described [38], [39] and cloned into a retrovirus vector pMXs to generate pMXs-AmCyan-hGeminin (1/110) and pMXs-IP-mCherry-hCdt1 (30/120). All constructs were verified by DNA sequencing. The pcDNA3-based vectors for Myc-CDH1 or Myc-CDC20 were provided by Dr. Masatoshi Fujita (Kyushu University, Fukuoka, Japan), pcDNA3-HA-Ubiquitin (WT) was from by Dr. Kazuo Sugamura (Miyagi Cancer Center Research Institute, Natori, Japan), pcDNA3.1-FLAG vectors for Skp2 and β-Trcp1/2 were from Dr. Kazuhiro Iwai (Osaka University, Suita, Japan), pcDNA3.1-FLAG-Fbw7 was from Dr. Kei-ichi Nakayama (Kyushu University, Fukuoka, Japan), pCSII-MCS-based vectors for AmCyan-hGeminin (1/110) and mCherry-hCdt1 (30/120) were from Dr. Atsushi Miyawaki (RIKEN, Wako, Japan), and the p3XFLAG-CMV10-XIAP was from Dr. Mikihiko Naito (National Institute of Health Science, Tokyo, Japan).

### Transfection and infection

Transfection and infection were performed as described previously [40]. Briefly, Plat-E cells (2×10^6^ cells) were seeded onto 100-mm dishes one day before the transfection. Cells were transfected by the calcium phosphate method. After 24 hr, the retroviral supernatant was collected and then used for infection of target cells.

### Time-lapse imaging

NIH3T3 cells transduced with Fucci2.1 indicators (pMXs-AmCyan-hGeminin (1/110) and pMXs-IP-mCherry-hCdt1 (30/120)) together with MgcRacGAP-mVenus were seeded onto 35-mm glass-bottom dishes in DMEM/10%FCS. Cells were subjected to time-lapse imaging using LCV100 (Olympus, Tokyo, Japan) with an objective lens ×40. More details were as previously given [38], [39].

### Immunoprecipitation and Western blotting

Immunoprecipitation (IP) and Western blotting (WB) were performed as described previously [13]. Protein concentrations in the lysate were determined, and samples were standardized to equal concentrations of protein. Equal amounts of total protein were loaded in each lane. Anti-Flag (M2), anti-α Tubulin (B-5-1-2) antibody (Ab) was from Sigma-Aldrich (St.Louis, USA) and anti-MgcRacGAP goat polyclonal (ab2270) Ab was from Abcam (Cambridge, UK). Anti-MgcRacGAP rabbit polyclonal Ab was generated as described previously [13]. Anti-cMyc (9E10) and anti-HA (12CA5) Ab were from Roche Applied Science (Penzberg, Germany).

### Synchronization of the cell cycle

Cells were synchronized in the metaphase by adding 40 ng/ml nocodazole (NDZ: Sigma-Aldrich, St. Louis, USA) for 12 hr or in the G0/1 phase by serum starvation for 12 hr. The cells were then washed and released in fresh medium. DNA content was analyzed by flow cytometry following propidium iodide staining.

### *In vivo* ubiquitination assay

Using the calcium phosphate method, 293T cells were transfected with 10 µg of pcDNA3-HA-Ubiquitin (WT) and 20 µg of pME18S-MgcRacGAP-Flag. After 12 hr, the medium was exchanged with fresh medium containing with 20 µM MG132. Then the cells were collected 24 hr after transfection.

### CDH1-mediated degradation

293T cells were transfected with 10 µg of pMXs-IG-Flag-tagged-WT or mutants of MgcRacGAP and 10 µg of pcDNA3-Myc-CDH1 expression vectors by the Ca phosphate method. After 36 hr, the medium was exchanged with fresh medium with or without 20 µM MG132. Then the cells were collected 48 hr after transfection.

### Flow cytometric analysis

Flow cytometric analysis was performed with FACSCalibur (BD Biosciences, San Jose, USA) equipped with FlowJo Version 7.2.4 software (Tree Star, Ashland, USA).

### Gene expression analysis

Gene expression analysis by qPCR was done as described elsewhere [41]. Briefly, total RNA was extracted using TRIzol (Invitrogen, Carlsbad, CA). cDNA was prepared with a High Capacity cDNA Reverse Transcription Kit (Life Technologies, Carlsbad, USA). Real-time reverse transcription–polymerase chain reaction (RT-PCR) was performed using a Rotor-Gene Q (QIAGEN, Hilden, Germany) and SYBR Premix EX Taq (TAKARA, Kyoto, Japan). All samples were independently analyzed at least three times. The following primer pairs were used: 5′- AACAGGCGCGGCGTGTTC -3′ (forward) and 5′- ATGAAGCGGTCACCATGC -3′ (reverse) for *Cdh1*; 5′- ATGTGTCCGTCGTGGATCTGA -3′ (forward) and 5′- TTGAAGTCGCAGGAGACAACCT -3′ (reverse) for *Gapdh*.

### Immunocytochemistry

Immunocytochemistry were performed as described previously [13].

### Statistical analysis

Statistical significance was calculated using the Student *t*-test for independent variables. *P* values<0.01 were considered statistically significant.

### Ethics statement

The mice were maintained and mated in the institutional animal facility according to the guidelines of the University of Tokyo. The experimental procedures in this study were approved by the Committee for Animal Experiments in the Institute of Medical Science University of Tokyo (approval number is PH10–14). All surgery was performed under sodium pentobarbital anesthesia, and all efforts were made to minimize suffering.

## Results

### MgcRacGAP is degraded in a cell cycle-dependent manner by the ubiquitin-dependent pathway during G0/1

**Figure 1:**
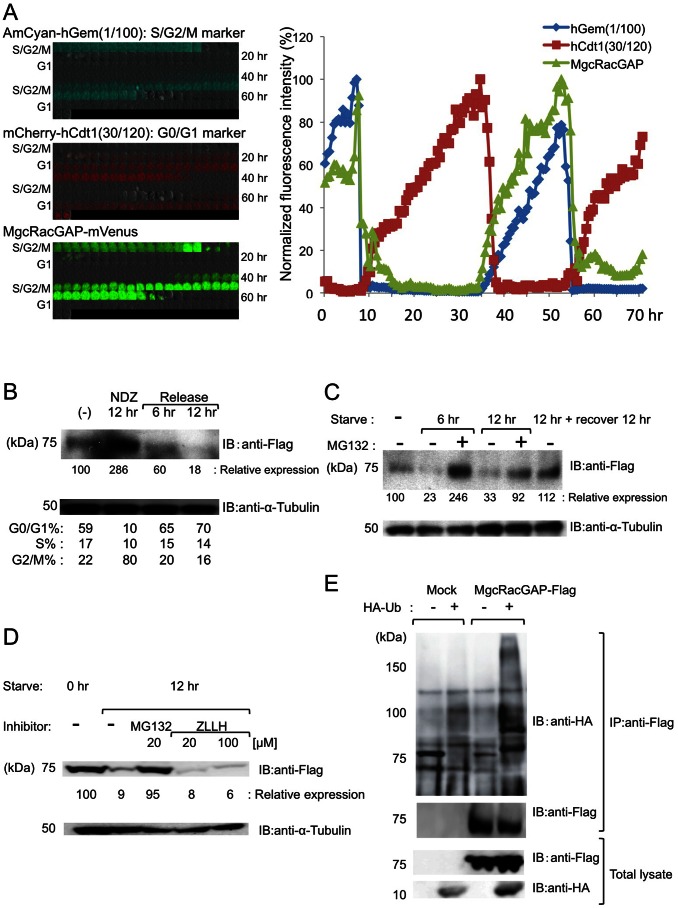
MgcRacGAP is degraded in a cell cycle-dependent manner by the ubiquitin-proteasome pathway. (A) NIH3T3 cells transduced with Fucci2.1 and MgcRacGAP-mVenus were analyzed by time-lapse imaging. Cell cycle-dependent changes in fluorescence of AmCyan-hGeminin(1/110), mCherry-hCdt1(30/120) and MgcRacGAP-mVenus (left panel). Temporal profiles of the normalized fluorescence intensities of AmCyan-hGeminin(1/110), mCherry-hCdt1(30/120) and MgcRacGAP-mVenus are shown in right panel. (B) NIH3T3 cells transduced with MgcRacGAP-Flag were treated with 40 ng/ml nocodazol (NDZ), then released in fresh medium and cultured for the indicated time period. Expression levels of MgcRacGAP-Flag (upper panel) and cell cycle status (lower panel) were analyzed. (C) NIH3T3 cells transduced with MgcRacGAP-Flag with or without serum starvation (6, 12 hr) or with serum starvation+ re-addition, in the absence (−) or presence (+) of 20 µM MG132. (D) Serum-starved NIH3T3 cells transduced with MgcRacGAP-Flag in the absence (−) or presence (+) of 20 µM MG132 or ZLLH were subjected to Western blotting. Relative band intensities of MgcRacGAP were calculated by densitometry analysis and normalized to α Tubulin. (E) The results of in vivo ubiquitination assay are shown. 293T cells were transfected with pME18S or pME18S-MgcRacGAP together with pcDNA3 or pcDNA3-HA-Ubiquitin (WT) and were analyzed by Western blotting. All of the results shown here are the representative of three independent experiments.

To investigate the changes of MgcRacGAP protein levels in real time, imaging studies with a cell cycle indicator system, Fucci, were performed. mVenus was fused to the C terminus of MgcRacGAP (MgcRacGAP-mVenus), and this fusion protein was retrovirally transduced to NIH3T3 cells together with two Fucci2.1 indicators (AmCyan-hGeminin (1/110) and mCherry-hCdt1 (30/120)) [38], [39]. MgcRacGAP-mVenus signals were strongly detected in the cells of the S/G2/M phase, but they were sharply decreased when the cells entered the G1 phase (Figure 1A and Movie S1). Flag-tagged MgcRacGAP (MgcRacGAP-Flag) was stably expressed in NIH3T3 cells. The cells were synchronized in the M-phase with 12 hours of NDZ treatment and then released into fresh medium and cultured for 6 or 12 hours. The cells were then analyzed for MgcRacGAP-Flag expression and cell cycle status. A maximal level of MgcRacGAP expression was observed in the M phase. Following the release, MgcRacGAP decreased and reached a minimal level in the G0/1 phase (Figure 1B). To investigate its expression in the G0/1 phase more precisely, the transduced NIH3T3 cells were serum-starved for 6 or 12 hours with or without re-addition of serum. More than 80% of the starved cells were in the G0/1 phase while only about 50–60% of the non-starved or released cells were found there (data not shown). As shown in Figure 1C, significantly reduced levels of MgcRacGAP proteins were observed in the starved compared with the non-starved cells. Expression of MgcRacGAP was recovered after serum re-addition or maintenance in serum-deprived medium in the presence of MG132, a proteasome inhibitor. Since MG132 is also capable of inhibiting the calpine pathway in addition to the proteasomal pathway, the effects of inhibiting this pathway were analyzed. Treatment with ZLLH, a calpine inhibitor, did not prevent the destruction of MgcRacGAP, while treatment with MG132 efficiently prevented its destruction (Figure 1D). Thus, degradation of MgcRacGAP is mainly mediated by proteasome. Similar results were also observed in the 293T cells (data not shown). In fact, co-expression of MgcRacGAP with HA-tagged Ubiquitin (WT) (HA-Ub) has revealed the ubiquitination of MgcRacGAP in 293T cells (Figure 1E).

These results indicate that MgcRacGAP is degraded in a cell cycle-dependent manner by the ubiquitin-proteasome pathway during the G0/1 phase.

### MgcRacGAP destruction is mediated by an E3 ligase APC^CDH1^

**Figure 2:**
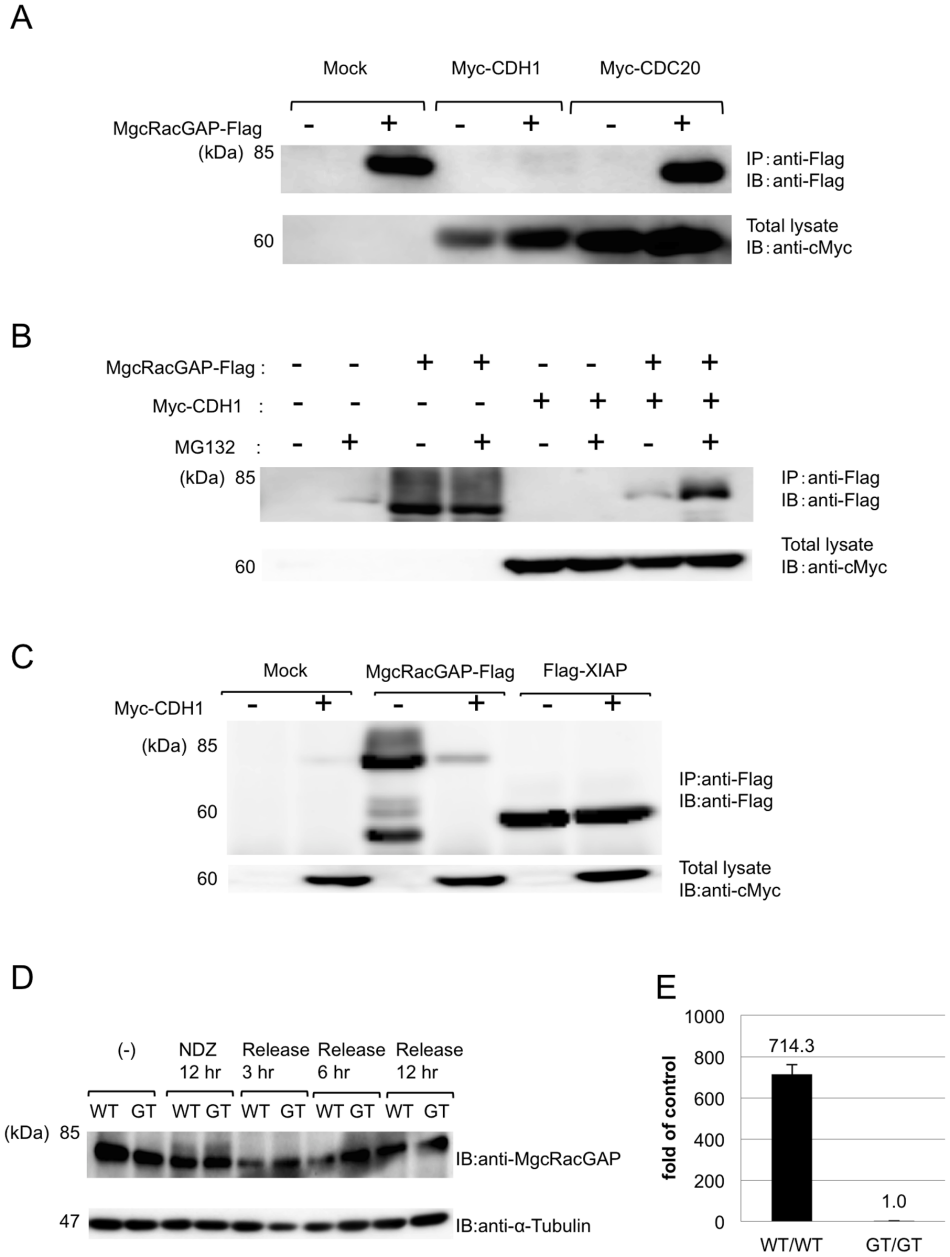
MgcRacGAP destruction is mediated by E3 ligase APC^CDH1^. (A) 293T cells co-transfected with pMXs-IG (−) or pMXs-IG-MgcRacGAPFlag (+), together with pcDNA3, or pcDNA3-Myc-CDH, or pcDNA3-Myc-CDC20, (B) 293T cells transfected with mock or MgcRacGAP-Flag, together with pcDNA3, or pcDNA3-Myc-CDH1 in the presence or absence of 20 µM MG132, and (C) 293T cells transfected with mock or MgcRacGAP-Flag or 3XFLAG-XIAP, together with pcDNA3 (−) or pcDNA3-Myc-CDH1 (+) were analyzed by Western blotting. (D) *Cdh1* GT/GT MEFs were treated by 40 ng/ml nocodazol (NDZ), then released in fresh medium and cultured for the indicated time period. Endogenous MgcRacGAP protein levels were analyzed by Western blotting. All of the results shown here are the representative of three independent experiments. (E) Relative levels of mRNA expression of *Cdh1* in wild type (WT/WT) and *Cdh1*-depleted (GT/GT) MEF cells were estimated by qPCR.

Protein ubiquitination is a critical step along the ubiquitin-proteasome pathway, and E3 ligase complexes mediate this step. APC/C is one of the E3 ligases that are activated in the M to G1 phase targeting their substrates for destruction. Considering the timing of MgcRacGAP destruction, it was possible that APC/C would mediate ubiquitination of MgcRacGAP. APC/C is activated by binding of either of the co-activators, CDH1 or CDC20. To examine APC/C's involvement in degradation of MgcRacGAP and to identify the E3 ligase for it, Myc-tagged CDH1 or CDC20 together with MgcRacGAP-Flag were co-transduced to 293T cells. MgcRacGAP proteins were profoundly decreased in CDH1-transduced cells but not in CDC20-transduced cells when compared to control cells (Figure 2A). Treatment with MG132 counteracted the effects of CDH1 expression (Figure 2B). To confirm the specificity of this reaction, we conducted a similar experiment using the cells transduced with Flag-tagged XIAP, which is regulated by ubiquitin-proteasome pathway but is not a target of APC^CDH1^, with or without co-expression of Myc-CDH1. CDH1 expression did not affect XIAP levels (Figure 2C). To study the interaction between MgcRacGAP and CDH1, MgcRacGAP-Flag and Myc-CDH1 were co-transfected in 293T cells. MgcRacGAP weakly bound to CDH1 (Figure S1). MEF derived from *Cdh1* GT/GT mice is useful for analysis of its substrates [37]. Knock-out of *Cdh1* resulted in accumulation of MgcRacGAP proteins in the G1 phase (Figures 2D and 2E). These results clearly demonstrate that degradation of MgcRacGAP in the G1 phase is mediated by APC^CDH1^.

### A C-terminal region, AA537–632, of MgcRacGAP contains a degron

**Figure 3:**
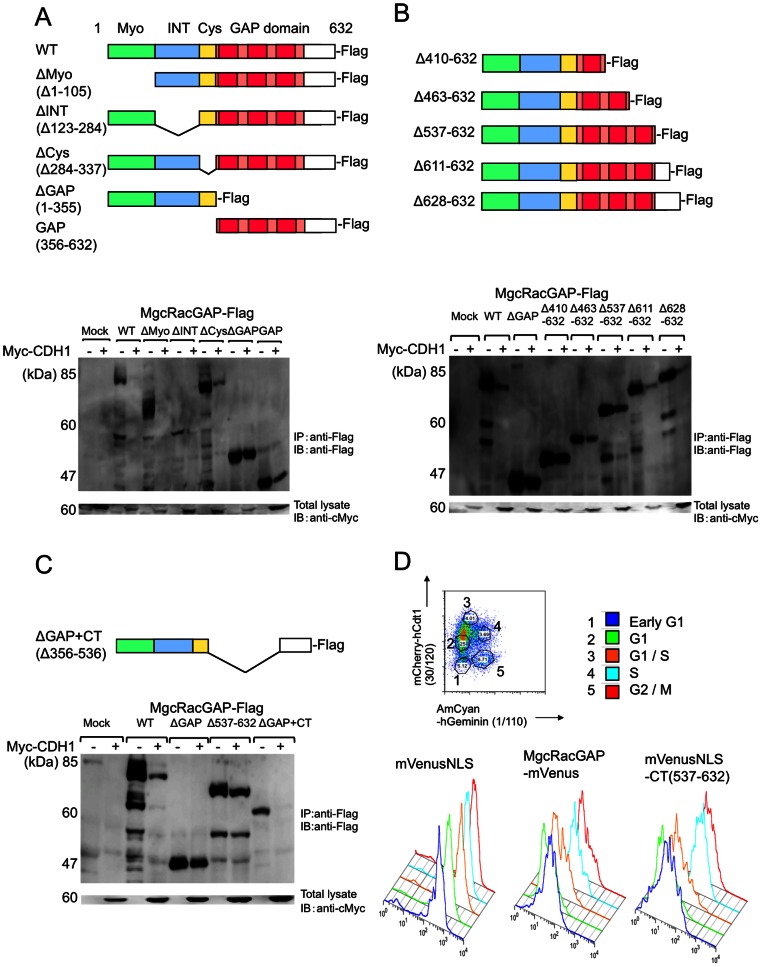
The C-terminal region of MgcRacGAP, AA 537–632, contains its degron. (A) WT and deletion mutants of MgcRacGAP are shown in the upper panel. Myo: myosine-like domain, Int: Internal domain, Cys: cystein-rich domain, GAP: GTPase-activating domain. The lower panel shows the result of Western blotting of 293T cells co-transfected with mock or WT or deletion mutant of MgcRacGAP-Flag, together with pcDNA3 (−) or pcDNA3-Myc-CDH1 (+). (B) WT and C-terminal deletion mutants of MgcRacGAP are shown in the upper panel. The lower panel shows the result of Western blotting of the 293T cells co-transfected with mock or WT or the deletion mutants of MgcRacGAP-Flag, together with pcDNA3 (−) or pcDNA3-Myc-CDH1 (+). (C) The upper panel shows the schema of MgcRacGAP deletion mutants, ΔGAP+CT. The lower panel shows the result of Western blotting of the 293T cells co-transfected with mock or WT or the deletion mutants of MgcRacGAP-Flag, together with pcDNA3 (−) or pcDNA3-Myc-CDH1 (+). (D) NIH3T3 cells transduced with mVenusNLS or MgcRacGAP-mVenus or mVenusNLS-CT together with Fucci 2.1 probes (mCherry-hCdt1 (1/30) and AmCyan-hGeminin (1/110)) were analyzed with FACS. The signal intensity of mVenus in each cell cycle phase is described. The results shown here represent three independent experiments.

To identify the region required for CDH1-mediated destruction, Flag-tagged WT or deletion mutants lacking any of the functional domains in MgcRacGAP were transiently expressed in 293T cells with or without Myc-CDH1. The deletion mutant lacking the GAP domain and the C terminus of MgcRacGAP (ΔGAP) was resistant to CDH1-mediated destruction. On the other hand, WT and the other deletion mutants, including the mutant with N-terminal truncation (GAP), were prone to CDH1-mediated destruction (Figure 3A).

This result indicated that the degron of MgcRacGAP is located within either the GAP domain or the C-terminal part of MgcRacGAP. To narrow down the region for CDH1-mediated destruction, we next generated mutants with several lengths of deletion in the C-terminal region — Δ410–632, Δ463–632, Δ537–632, Δ611–632, and Δ628–632 (Figure 3B). Among the mutants analyzed here, the mutant truncated at the position of AA536 (Δ537–632) or the mutants with longer C-terminal truncation (Δ410–632, Δ463–632) resisted destruction, while the mutants with shorter C-terminal truncation (Δ611–632, Δ628–632) were prone to destruction (Figure 3B, lower panel). Imaging studies using mCherry-tagged WT and Δ537–632 demonstrated that mutant Δ537–632 was nondegradable in the G1 phase (Figure S2A, Movie S2 and S3). By immunocytochemistry, Flag tagged WT and Δ537–632 was localized to the nucleus (Figure S2B). Although the function of AA537–632 is not known, the RhoGAP activity of Δ537–632 was comparable to that of WT MgcRacGAP (data not shown). These observations indicate that the residues between AA537–632 (CT) are required for CDH1-mediated destruction and contain the degron but do not contribute to the GAP activity.

The CT region of AA537–632, which starts from the position closest to the C-terminal end of the GAP domain, was fused to a destruction-resistant mutant, ΔGAP to form another mutant, ΔGAP+CT. To examine whether the CT region is sufficient for targeting MgcRacGAP destruction, ΔGAP or ΔGAP+CT was transduced to 293T cells with or without Myc-CDH1. As expected, ΔGAP+CT was degraded in the presence of CDH1, but ΔGAP was not (Figure 3C).

**Figure 4:**
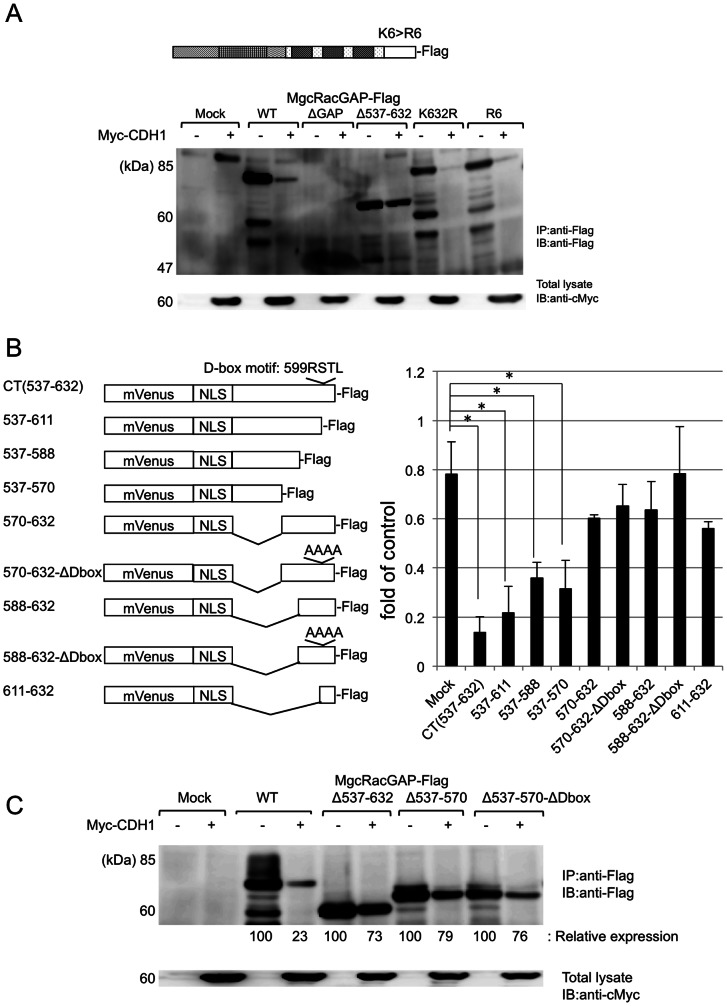
The degron of MgcRacGAP contains a putative D-box and an essential region for destruction. (A) The upper panel shows the schema of fusion proteins between mVenusNLS and full-length or partial fragments of the CT region of MgcRacGAP. The lower panel shows the result of FACS analysis of 293T cells co-transfected with each fusion protein, together with pcDNA3 or Myc-CDH1. Inhibition rate is calculated by the ratio of (the % of mVenus (+) cells in CDH1 transfectants)/(the % of mVenus (+) cells in mock transfectants). The results shown are the means of three independent experiments, and the error bars indicate the standard deviation of the mean (* P<0.01). (B) 293T cells co-transfected with mock or WT or the deletion mutants (Δ537–632, Δ537–570, Δ537–570-ΔDbox) of MgcRacGAP-Flag, together with pcDNA3 (−) or pcDNA3-Myc-CDH1 (+). Relative band intensities of MgcRacGAP were calculated by densitometry analysis and normalized to α Tubulin.

We also fused the CT region to mVenus (mVenus-CT) to ascertain whether this region is sufficient to induce degradation of proteins other than MgcRacGAP. However, the levels of this fusion protein were only slightly diminished in the G1 phase compared with the S/G2/M phase (data not shown). Most mVenus-CT proteins were expressed in the cytoplasm, while most endogenous and transduced MgcRacGAP proteins were observed in the nucleus. It has been reported that nuclear transport of Ect2 is required for its destruction mediated by Cdh1 [36], and it is possible that the degron of MgcRacGAP is also functional only in the nucleus. So we next replaced mVenus of this fusion protein with mVenus with the nuclear localization signal (mVenusNLS) to produce mVenusNLS-CT. This fusion protein was retrovirally transduced to NIH3T3 cells together with Fucci2.1 indicators. mVenusNLS-CT expression was changed in the cell-cycle dependent manners as well as MgcRacGAP-mVenus expression (Figure 3D). The co-expression of Myc-CDH1 and mVenusNLS fused to the C-terminal fragment (AA537–632) of MgcRacGAP revealed that mVenusNLS-CT was targeted for destruction by CDH1 (Figure 4B). Taken together, the present results clearly show that the residues between AA537–632 of MgcRacGAP are necessary and sufficient for the destruction of MgcRacGAP mediated by CDH1.

### The degron of MgcRacGAP contains a putative D-box and an essential region for destruction

Degron, the minimal region with a destruction signal, usually contains recognition sites for E3 ligase, ubiquitination sites, or initiation sites for ubiquitin chain elongation [42]. Several recognition motifs for APC/C have been identified in target proteins of APC/C, such as the D-box, the KEN box, and the TEK box [43]. Sequence analysis revealed the existence of a putative D-box, AA599RSTLAA602, within the CT region. We replaced the four residues of the putative D-box with four alanines to make the D-box deficient mutant, ΔDbox. However, co-expression of ΔDbox with Myc-CDH1 revealed that the ΔDbox was degraded as had WT-MgcRacGAP been (data not shown). The result indicated that this putative D-box is not functional in MgcRacGAP. As is the case with several other APC/C target proteins such as Cyclin B or Ect2, it is possible that the disrupting the D-box alone is not enough to prevent destruction.

Lysine is known to be a residue usually linked to ubiquitin, and there are six lysine residues within the CT region (K571, K587, K604, K612, K614, and K632). To identify the ubiquitination sites in the CT region, we replaced one of each lysine residue or all six (R6) residues with arginine. However, Myc-CDH1 was able to degrade all the mutants as was the case for WT-MgcRacGAP (Figure 4A and data not shown).

To find a region in the CT region critical for the destruction of MgcRacGAP, full-length or partial fragments of the CT region were fused to mVenusNLS (Figure 4B, left panel) and transduced to 293T cells with or without transfection of Myc-CDH1. After the transfection, degradation of the fusion proteins was assessed by the intensity of fluorescence. This co-expression study revealed that the residues between AA537 and 570 were sufficient to induce the CDH1-dependent efficient destruction of the fusion protein (Figure 4B, right panel). Of note, a co-expression study using MgcRacGAP deletion mutants demonstrated that the mutants lacking either AA537–570 (Δ537–570) or AA537–570 and D-box (Δ537–570-ΔDbox) resisted CDH1-mediated destruction. Thus, the residues between AA537 and 570 were indispensable for the destruction of MgcRacGAP (Figure 4C).

## Discussion

MgcRacGAP plays distinct roles in regulating Rho family GTPases, depending on the cell cycle. In the interphase, MgcRacGAP is critical for the nuclear transport of STAT3/5 transcription factors, working as a Rac1-GAP [21]–[25]. In the telophase, MgcRacGAP is indispensable as a RhoA-GAP for the completion of cytokinesis [10]–[15]. In the metaphase, it is suggested that MgcRacGAP is important in the segregation of chromosomes, working as a Cdc42-GAP [18]. It is assumed that these pleiotropic functions of MgcRacGAP are controlled by elaborate mechanisms, including phosphorylation, subcellular localization and control of expression levels. The expression of MgcRacGAP is tightly regulated in a cell cycle-dependent manner; MgcRacGAP is highly expressed in the S/G2/M phase and sharply decreased in the late M to G1 phase [26]. One possible mechanism accounting for this decrease is degradation via the ubiquitin-proteasome pathway, as several other cell cycle regulators such as Geminin, Securin and Aurora A/B are repressed in the late M phase. APC^Cdh1^, APC^Cdc20^, and SCF^b-Trcp^ are E3 ligases responsible for ubiquitination and destruction of these molecules during the late M to G0/1 phase.

In the present study, we have demonstrated that MgcRacGAP is degraded during the late M to G1 phase by ubiquitin-dependent mechanisms (Figure 1 and Movie S1). We also demonstrated that MgcRacGAP is a novel target of a cell cycle-dependent ubiquitin ligase, APC^Cdh1^, and that genetic disruption of Cdh1 diminished the degradation of MgcRacGAP in the G0/1phase (Figure 2). Cdh1, a co-activator of APC/C complex, is activated during the late M to G1 phase. Most targets of APC^Cdh1^ are involved in the cell cycle, including Cdc20, Skp2, CyclinA/B, Cdc25, and Geminin [33]. Aurora B, a kinase for MgcRacGAP, and p190RhoGAP, a cell cycle-dependent RhoGAP, are well-known substrates of APC^Cdh1^ [35], [37]. It has recently been reported that Ect2, a RhoGEF involved in mitosis, is also a target of Cdh1 [36]. We were not able to completely exclude the possibility that E3 ligases other than APC^Cdh1^ regulate MgcRacGAP protein levels. However, our co-expression studies revealed that neither overexpression of Cdc20, another co-activator of APC/C complex (Figure 2), nor some of the effector subunits of SCF complexes such as Skp2, Fbw7, and β-Trcp1/2 (data not shown), changed MgcRacGAP protein levels.

These results indicate that APC^CDH1^ is a major E3 ligase for MgcRacGAP. Experimental results using deletion mutants of MgcRacGAP and the fusion proteins with mVenusNLS indicated that the C-terminal residues of MgcRacGAP, AA537–632 (CT), contain its degron (Figures 3 and 4). This CT region of MgcRacGAP contains six lysine residues, which might have contained ubiquitination sites. However, replacing of all or any lysine residues failed to prevent MgcRacGAP destruction (Figure 4A and data not shown). The lysine residue for ubiquitination may exist outside of the CT region, or N-terminal ubiquitination using residues other than lysine may also contribute to the ubiquitination of MgcRacGAP, as is the case for p21 [44], [45].

Many substrates of APC/C contain a recognition motif for E3 ligases such a D-box, KEN, TEK, GxEN, A-box, or O-box [43], while some of the substrates have no known recognition motifs [46]. The CT region contains one putative D-box of the type RxxL, AA599 RSTL AA602. However, deleting the D-box alone did not affect the destruction of MgcRacGAP (Figure 4C). As reported previously, a protein motif adjacent to the D-box, called the ubiquitin chain initiation motif (IM), is important for efficient ubiquitination of several substrates by APC/C, including Securin, Geminin, and Ect2 [36], [47]. IM has not been well characterized so far, and we were not able to identify any residues in the CT region similar to the IM of other molecules. However, sequence analysis of MgcRacGAP with pestfind (http://emboss.bioinformatics.nl/cgi-bin/emboss/pestfind) [48] indicated the existence of a PEST domain-like structure in the residues between AA530–554, close to the putative D-box (Table 1). In addition, studies with fusion proteins indicated that most of the 25 residues are included in the critical region for destruction between AA537 and AA570. As reported, the charged residues within an IM are required for its function [47], and interestingly, there are six charged residues in the PEST domain-like region. These observations indicate that this region may possess functions similar to an IM, though further study is reqired to confirm this hypothesis.

**Table 1:** PEST domains of MgcRacGAP.

PEST-score	Sequence	Residues
−1.19	KTTVTVPNDGGPIEAVSTIETVPYWTR	218–244
−2.06	KTGTLQPWNSDSTLNSR	248–264
+1.56	RTETDSVGTPQSNGGMR	269–285
−17.02	KPESCVPCGK	296–305
−16.00	KIGEGMLADFVSQTSPMIPSIVVH	345–368
−8.66	RAFMEAAEITDEDNSIAAMYQAVGELPQANR	440–470
−11.43	HAVPNPDPVTMLQDIK	507–522
−12.52	RLLSLPLEYWSQFMMVEQENIDPLH	530–554*
−4.67	HVIENSNAFSTPQTPDIK	554–571*
−11.19	KVSLLGPVTTPEH	571–583

Protein sequence of MgcRacGAP analyzed and PEST-scores were calculated by pestfind.

The positive value of PEST-score indicated the possible PEST sequence.

The negative value of PEST-score also indicated the weakly possible PEST sequence.

* Include essential region (537–570).

In the present study, we demonstrated a new way to regulate MgcRacGAP expression and the function of its C-terminal region (AA537–570) as a degron. Our findings not only reveal a novel mechanism to control MgcRacGAP but also help expand the knowledge of protein degradation via APC^CDH1^.

## Supporting Information

Figure S1 **MgcRacGAP binds to CDH1.** 293T cells co-transfected with mock or MgcRacGAP-Flag, together with pcDNA3 (−) or pcDNA3-Myc-CDH1 (+).
(TIF)

Figure S2 **MgcRacGAP (Δ537–632)-mCherry is not degraded in the G0/G1 phase and localized to the nucleus.** (A) NIH3T3 cells transduced with pMXs-IRES-Puro^r^-MgcRacGAP (WT)-mCherry or MgcRacGAP (Δ537–632)-mCherry were stained with Hoechest 33342 and analyzed with FACS (top panel). DAPI staining was subjected to microscopic analysis by Olympus IX71 and Fluoview with X 100 Objective lens (Olympus, Tokyo, Japan) (middle: normal conditions, bottom: serum starvation). (B) NIH3T3 cells transduced with pMXs-IRES-Puro^r^-MgcRacGAP (WT)-Flag or MgcRacGAP (Δ537–632)-Flag were stained with DAPI and anti-Flag (M2) and view with Olympus IX71 and Fluoview with X 100 Objective lens.
(TIF)

Movie S1 **Cell cycle-dependent degradation of MgcRacGAP.** NIH3T3 cells transduced with Fucci2.1 probes (AmCyan-hGeminin (1/110) and mCherry-hCdt1 (30/120)) (left panel) together with MgcRacGAP-mVenus (right panel) were analyzed with Olympus LCV-110 with X 40 Objective lens (Olympus, Tokyo, Japan) at 30 min intervals.
(MP4)

Movie S2 **MgcRacGAP (WT)-mCherry is degraded in the G0/G1 phase.** NIH3T3 cells transduced with pMXs-IRES-Puro^r^-MgcRacGAP (WT)-mCherry were analyzed with Nikon Biostation with X 20 objective lens at 15 min intervals.
(MP4)

Movie S3 **MgcRacGAP (Δ537–632)-mCherry is not degraded in the G0/G1 phase.** NIH3T3 cells transduced with pMXs-IRES-Puro^r^-MgcRacGAP (Δ537–632)-mCherry were analyzed with Nikon Biostation with X 20 objective lens at 15 min intervals.
(MP4)

Protocol S1 **Hoechst staining and cell cycle analysis.** NIH3T3 cells transduced with MgcRacGAP (WT)-mCherry or MgcRacGAP (Δ537–632)-mCherry were stained with 5 µg/ml Hoechst 33342 (Invitrogen, Carlsbad, CA) and 20 µg/ml verapamil (Sigma-Aldrich, St. Louis, USA). After incubation for 30 min, DNA content of mCherry (+) cells was analyzed by FACSAria (BD Biosciences, San Jose, USA).
(DOC)

## References

[1] Touré A, Dorseuil O, Morin L, Timmons P, Jégou B, et al (1998) MgcRacGAP, a new human GTPase-activating protein for Rac and Cdc42 similar to Drosophila rotundRacGAP gene product, is expressed in male germ cells. J Biol Chem 273: 6019–6023. 9497316 10.1074/jbc.273.11.6019

[2] Lee JS, Kamijo K, Ohara N, Kitamura T, Miki T (2004) MgcRacGAP regulates cortical activity through RhoA during cytokinesis. Exp Cell Res 293: 275–282. 14729465 10.1016/j.yexcr.2003.10.015

[3] Kaibuchi K, Kuroda S, Amano M (1999) Regulation of the cytoskeleton and cell adhesion by the Rho family GTPases in mammalian cells. Annu Rev Biochem 68: 459–486. 10872457 10.1146/annurev.biochem.68.1.459

[4] Etienne-Manneville S, Hall A (2002) Rho GTPases in cell biology. Nature 420: 629–635. 12478284 10.1038/nature01148

[5] Mishima M, Glotzer M (2003) Cytokinesis: a logical GAP. Curr Biol 13: R589–591. 12906808 10.1016/s0960-9822(03)00521-9

[6] Piekny A, Werner M, Glotzer M (2005) Cytokinesis: welcome to the Rho zone. Trends Cell Biol 15: 651–658. 16243528 10.1016/j.tcb.2005.10.006

[7] D'Avino PP, Glover DM (2009) Cytokinesis: mind the GAP. Nat Cell Biol 11: 112–114. 19188915 10.1038/ncb0209-112

[8] Glotzer M (2009) Cytokinesis: GAP gap. Curr Biol 19: R162–165. 19243691 10.1016/j.cub.2008.12.028 PMC8875700

[9] Minoshima Y, Kawashima T, Hirose K, Tonozuka Y, Kawajiri A, et al (2003) Phosphorylation by aurora B converts MgcRacGAP to a RhoGAP during cytokinesis. Dev Cell 4: 549–560. 12689593 10.1016/s1534-5807(03)00089-3

[10] Mishima M, Kaitna S, Glotzer M (2002) Central spindle assembly and cytokinesis require a kinesin-like protein/RhoGAP complex with microtubule bundling activity. Dev Cell 2: 41–54. 11782313 10.1016/s1534-5807(01)00110-1

[11] Lekomtsev S, Su KC, Pye VE, Blight K, Sundaramoorthy S, et al (2012) Centralspindlin links the mitotic spindle to the plasma membrane during cytokinesis. Nature 492: 276–279. 23235882 10.1038/nature11773

[12] Yamada T, Hikida M, Kurosaki T (2006) Regulation of cytokinesis by mgcRacGAP in B lymphocytes is independent of GAP activity. Exp Cell Res 312: 3517–3525. 16959247 10.1016/j.yexcr.2006.07.026

[13] Hirose K, Kawashima T, Iwamoto I, Nosaka T, Kitamura T (2001) MgcRacGAP is involved in cytokinesis through associating with mitotic spindle and midbody. J Biol Chem 276: 5821–5828. 11085985 10.1074/jbc.M007252200

[14] Zhao WM, Fang G (2005) MgcRacGAP controls the assembly of the contractile ring and the initiation of cytokinesis. Proc Natl Acad Sci U S A 102: 13158–13163. 16129829 10.1073/pnas.0504145102 PMC1201590

[15] Jantsch-Plunger V, Gönczy P, Romano A, Schnabel H, Hamill D, et al (2000) CYK-4: A Rho family gtpase activating protein (GAP) required for central spindle formation and cytokinesis. J Cell Biol 149: 1391–1404. 10871280 10.1083/jcb.149.7.1391 PMC2175131

[16] Van de Putte T, Zwijsen A, Lonnoy O, Rybin V, Cozijnsen M, et al (2001) Mice with a homozygous gene trap vector insertion in mgcRacGAP die during pre-implantation development. Mech Dev 102: 33–44. 11287179 10.1016/s0925-4773(01)00279-9

[17] Yamada T, Kurosaki T, Hikida M (2008) Essential roles of mgcRacGAP in multilineage differentiation and survival of murine hematopoietic cells. Biochem Biophys Res Commun 372: 941–946. 18541143 10.1016/j.bbrc.2008.05.170

[18] Oceguera-Yanez F, Kimura K, Yasuda S, Higashida C, Kitamura T, et al (2005) Ect2 and MgcRacGAP regulate the activation and function of Cdc42 in mitosis. J Cell Biol 168: 221–232. 15642749 10.1083/jcb.200408085 PMC2171585

[19] Miller AL, Bement WM (2009) Regulation of cytokinesis by Rho GTPase flux. Nat Cell Biol 11: 71–77. 19060892 10.1038/ncb1814 PMC2677303

[20] Lagana A, Dorn JF, De Rop V, Ladouceur AM, Maddox AS, et al (2010) A small GTPase molecular switch regulates epigenetic centromere maintenance by stabilizing newly incorporated CENP-A. Nat Cell Biol 12: 1186–1193. 21102442 10.1038/ncb2129

[21] Kawashima T, Hirose K, Satoh T, Kaneko A, Ikeda Y, et al (2000) MgcRacGAP is involved in the control of growth and differentiation of hematopoietic cells. Blood 96: 2116–2124. 10979956

[22] Kitamura T, Kawashima T, Minoshima Y, Tonozuka Y, Hirose K, et al (2001) Role of MgcRacGAP/Cyk4 as a regulator of the small GTPase Rho family in cytokinesis and cell differentiation. Cell Struct Funct 26: 645–651. 11942621 10.1247/csf.26.645

[23] Tonozuka Y, Minoshima Y, Bao YC, Moon Y, Tsubono Y, et al (2004) A GTPase-activating protein binds STAT3 and is required for IL-6-induced STAT3 activation and for differentiation of a leukemic cell line. Blood 104: 3550–3557. 15284113 10.1182/blood-2004-03-1066

[24] Kawashima T, Bao YC, Nomura Y, Moon Y, Tonozuka Y, et al (2006) Rac1 and a GTPase-activating protein, MgcRacGAP, are required for nuclear translocation of STAT transcription factors. J Cell Biol 175: 937–946. 17178910 10.1083/jcb.200604073 PMC2064703

[25] Kawashima T, Bao YC, Minoshima Y, Nomura Y, Hatori T, et al (2009) A Rac GTPase-activating protein, MgcRacGAP, is a nuclear localizing signal-containing nuclear chaperone in the activation of STAT transcription factors. Mol Cell Biol 29: 1796–1813. 19158271 10.1128/MCB.01423-08 PMC2655612

[26] Seguin L, Liot C, Mzali R, Harada R, Siret A, et al (2009) CUX1 and E2F1 regulate coordinated expression of the mitotic complex genes Ect2, MgcRacGAP, and MKLP1 in S phase. Mol Cell Biol 29: 570–581. 19015243 10.1128/MCB.01275-08 PMC2612504

[27] Burkard ME, Maciejowski J, Rodriguez-Bravo V, Repka M, Lowery DM, et al (2009) Plk1 self-organization and priming phosphorylation of HsCYK-4 at the spindle midzone regulate the onset of division in human cells. PLoS Biol 7: e1000111. 19468302 10.1371/journal.pbio.1000111 PMC2680336

[28] Canman JC, Lewellyn L, Laband K, Smerdon SJ, Desai A, et al (2008) Inhibition of Rac by the GAP activity of centralspindlin is essential for cytokinesis. Science 322: 1543–1546. 19056985 10.1126/science.1163086 PMC2736296

[29] Ban R, Irino Y, Fukami K, Tanaka H (2004) Human mitotic spindle-associated protein PRC1 inhibits MgcRacGAP activity toward Cdc42 during the metaphase. J Biol Chem 279: 16394–16402. 14744859 10.1074/jbc.M313257200

[30] Ang XL, Harper JW (2004) Interwoven ubiquitination oscillators and control of cell cycle transitions. Sci STKE 2004: pe31. 15266102 10.1126/stke.2422004pe31

[31] Vodermaier HC (2004) APC/C and SCF: controlling each other and the cell cycle. Curr Biol 14: R787–796. 15380093 10.1016/j.cub.2004.09.020

[32] Nakayama KI, Nakayama K (2006) Ubiquitin ligases: cell-cycle control and cancer. Nat Rev Cancer 6: 369–381. 16633365 10.1038/nrc1881

[33] Qiao X, Zhang L, Gamper AM, Fujita T, Wan Y (2010) APC/C-Cdh1: from cell cycle to cellular differentiation and genomic integrity. Cell Cycle 9: 3904–3912. 20935501 10.4161/cc.9.19.13585 PMC3047751

[34] Littlepage LE, Ruderman JV (2002) Identification of a new APC/C recognition domain, the A box, which is required for the Cdh1-dependent destruction of the kinase Aurora-A during mitotic exit. Genes Dev 16: 2274–2285. 12208850 10.1101/gad.1007302 PMC186670

[35] Stewart S, Fang G (2005) Destruction box-dependent degradation of aurora B is mediated by the anaphase-promoting complex/cyclosome and Cdh1. Cancer Res 65: 8730–8735. 16204042 10.1158/0008-5472.CAN-05-1500

[36] Liot C, Seguin L, Siret A, Crouin C, Schmidt S, et al (2011) APC(cdh1) mediates degradation of the oncogenic Rho-GEF Ect2 after mitosis. PLoS One 6: e23676. 21886810 10.1371/journal.pone.0023676 PMC3158779

[37] Naoe H, Araki K, Nagano O, Kobayashi Y, Ishizawa J, et al (2010) The anaphase-promoting complex/cyclosome activator Cdh1 modulates Rho GTPase by targeting p190 RhoGAP for degradation. Mol Cell Biol 30: 3994–4005. 20530197 10.1128/MCB.01358-09 PMC2916440

[38] Sakaue-Sawano A, Kurokawa H, Morimura T, Hanyu A, Hama H, et al (2008) Visualizing spatiotemporal dynamics of multicellular cell-cycle progression. Cell 132: 487–498. 18267078 10.1016/j.cell.2007.12.033

[39] Sakaue-Sawano A, Ohtawa K, Hama H, Kawano M, Ogawa M, et al (2008) Tracing the silhouette of individual cells in S/G2/M phases with fluorescence. Chem Biol 15: 1243–1248. 19101468 10.1016/j.chembiol.2008.10.015

[40] Kitamura T, Onishi M, Kinoshita S, Shibuya A, Miyajima A, et al (1995) Efficient screening of retroviral cDNA expression libraries. Proc Natl Acad Sci U S A 92: 9146–9150. 7568090 10.1073/pnas.92.20.9146 PMC40941

[41] Watanabe-Okochi N, Kitaura J, Ono R, Harada H, Harada Y, et al (2008) AML1 mutations induced MDS and MDS/AML in a mouse BMT model. Blood 111: 4297–4308. 18192504 10.1182/blood-2007-01-068346

[42] Inobe T, Fishbain S, Prakash S, Matouschek A (2011) Defining the geometry of the two-component proteasome degron. Nat Chem Biol 7: 161–167. 21278740 10.1038/nchembio.521 PMC3129032

[43] Wäsch R, Robbins JA, Cross FR (2010) The emerging role of APC/CCdh1 in controlling differentiation, genomic stability and tumor suppression. Oncogene 29: 1–10. 19826416 10.1038/onc.2009.325 PMC3102600

[44] Bloom J, Amador V, Bartolini F, DeMartino G, Pagano M (2003) Proteasome-mediated degradation of p21 via N-terminal ubiquitinylation. Cell 115: 71–82. 14532004 10.1016/s0092-8674(03)00755-4

[45] Ciechanover A, Ben-Saadon R (2004) N-terminal ubiquitination: more protein substrates join in. Trends Cell Biol 14: 103–106. 15055197 10.1016/j.tcb.2004.01.004

[46] Cotto-Rios XM, Jones MJ, Busino L, Pagano M, Huang TT (2011) APC/CCdh1-dependent proteolysis of USP1 regulates the response to UV-mediated DNA damage. J Cell Biol 194: 177–186. 21768287 10.1083/jcb.201101062 PMC3144416

[47] Williamson A, Banerjee S, Zhu X, Philipp I, Iavarone AT, et al (2011) Regulation of ubiquitin chain initiation to control the timing of substrate degradation. Mol Cell 42: 744–757. 21700221 10.1016/j.molcel.2011.04.022 PMC3125540

[48] Rogers S, Wells R, Rechsteiner M (1986) Amino acid sequences common to rapidly degraded proteins: the PEST hypothesis. Science 234: 364–368. 2876518 10.1126/science.2876518

