# Correction: APC^CDH1^ Targets MgcRacGAP for Destruction in the Late M Phase

**DOI:** 10.1371/journal.pone.0089706

**Published:** 2014-03-05

**Authors:** The PLOS ONE Staff

The legend for Figure 4 was incorrect. It is corrected here.

**Figure 4:**
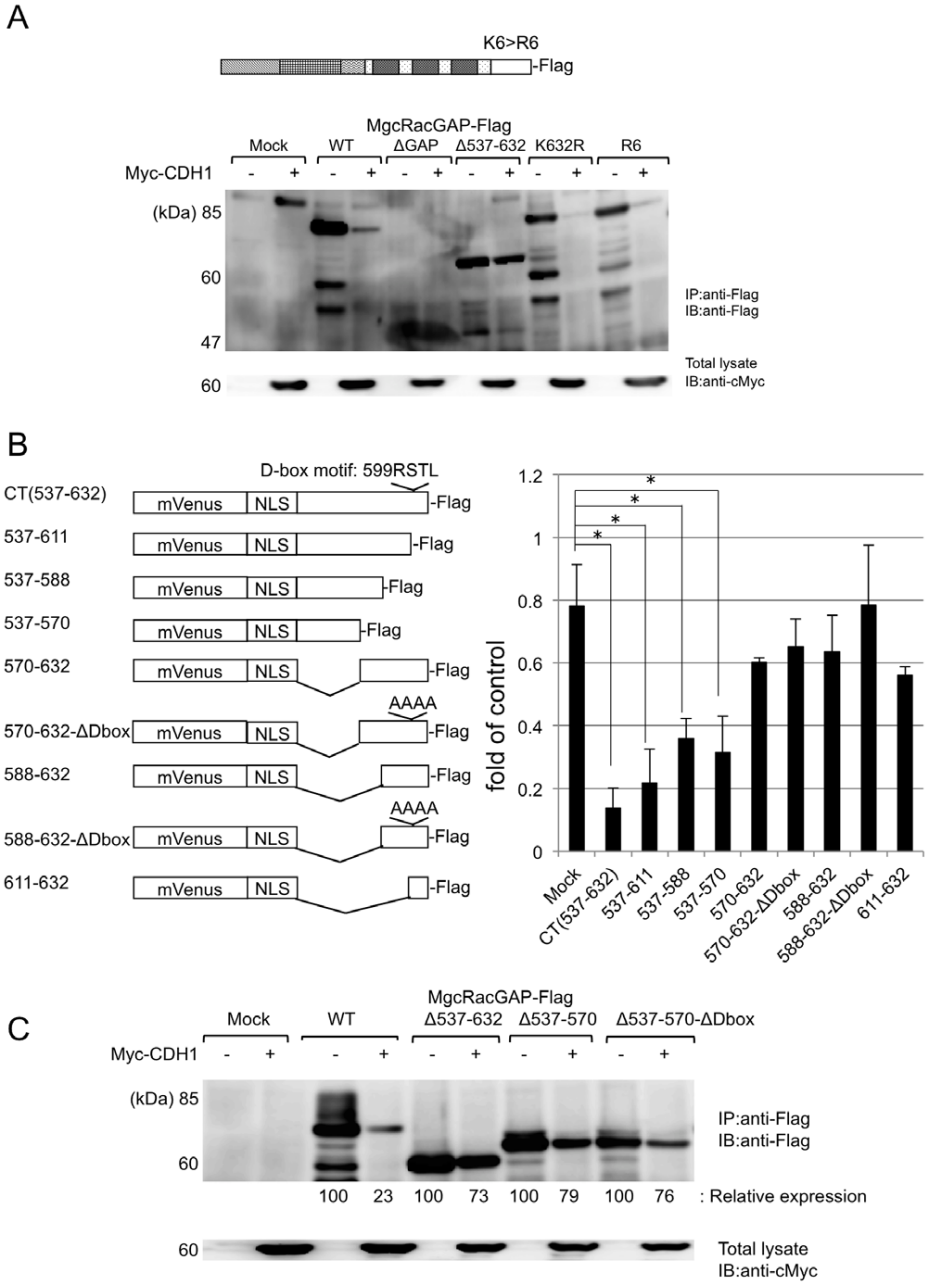
The degron of MgcRacGAP contains a putative D-box and an essential region for destruction. (A) The upper panel shows the schema of MgcRacGAP KR mutants. The lower panel shows the result of Western blotting of 293T cells co-transfected with mock or WT or deletion mutants (ΔGAP, Δ537-632) or KR(K632R, R6) mutants of MgcRacGAP-Flag, together with pcDNA3 (-) or pcDNA3 Myc-CDH1 (+). (B) The left panel shows the schema of fusion proteins between mVenusNLS and full-length or partial fragments of the CT region of MgcRacGAP. The right panel shows the result of FACS analysis of 293T cells co-transfected with each fusion protein, together with pcDNA3 or Myc-CDH1. Inhibition rate is calculated by the ratio of (the % of mVenus (+) cells in CDH1 transfectants)/ (the % of mVenus (+) cells in mock transfectants). The results shown are the means of three independent experiments, and the error bars indicate the standard deviation of the mean (* P <0.01). (C) 293T cells co-transfected with mock or WT or the deletion mutants (Δ537-632, Δ537-570, Δ537-570-ΔDbox) of MgcRacGAP-Flag, together with pcDNA3 (-) or pcDNA3-Myc-CDH1 (+). Relative band intensities of MgcRacGAP were calculated by densitometry analysis and normalized to α-Tubulin.

## References

[1] Nishimura K, Oki T, Kitaura J, Kuninaka S, Saya H, et al (2013) APC^CDH1^ Targets MgcRacGAP for Destruction in the Late M Phase. PLoS ONE 8(5): e63001 doi:10.1371/journal.pone.0063001 23696789 10.1371/journal.pone.0063001 PMC3656054

