# Supplementary figures and images for: APCCDH1 Targets MgcRacGAP for Destruction in the Late M Phase
Source: PLoS One. 2013 May 16;8(5):e63001. doi: 10.1371/journal.pone.0063001 (PMC3656054; doi:10.1371/journal.pone.0063001)

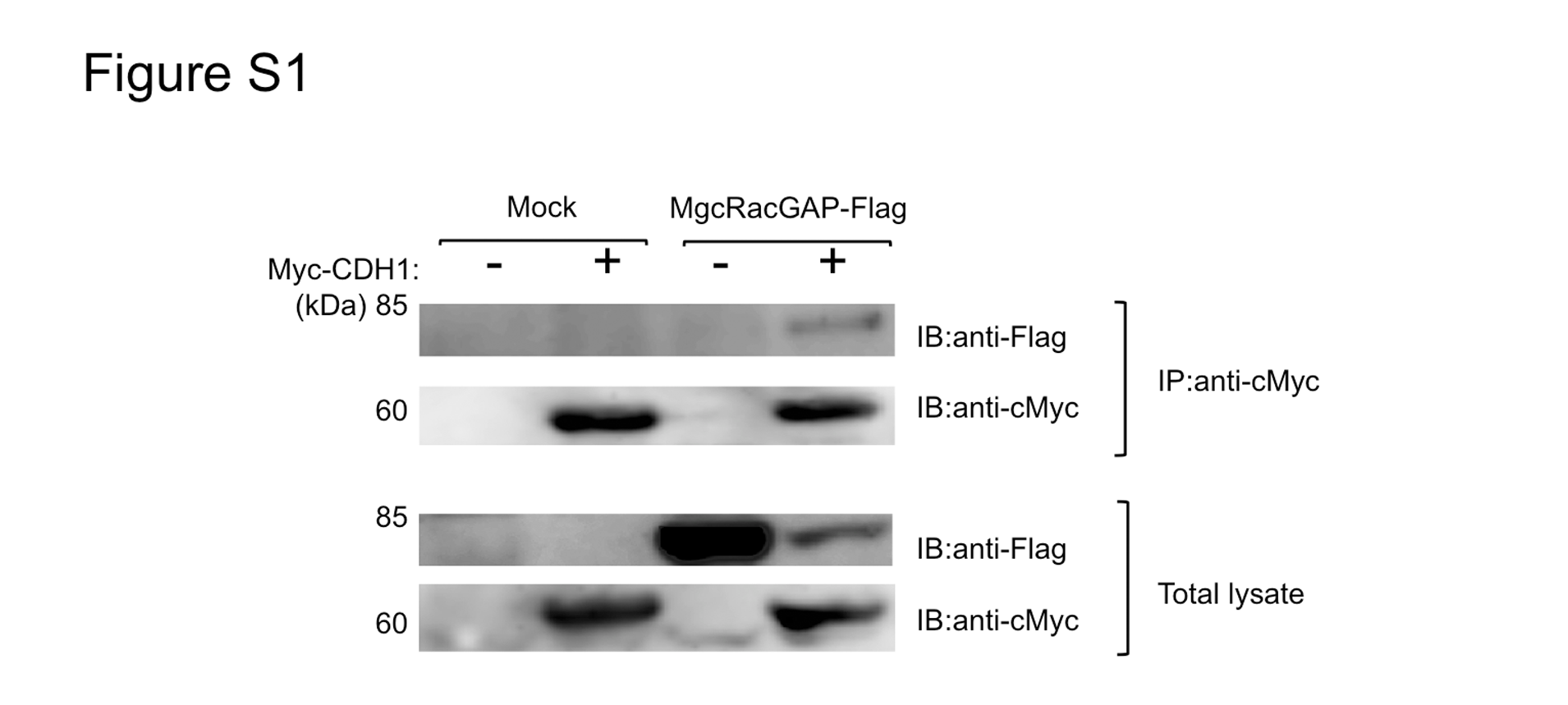

Supplement: Figure S1 — MgcRacGAP binds to CDH1. 293T cells co-transfected with mock or MgcRacGAP-Flag, together with pcDNA3 (−) or pcDNA3-Myc-CDH1 (+). (TIF) [file pone.0063001.s001.tif]

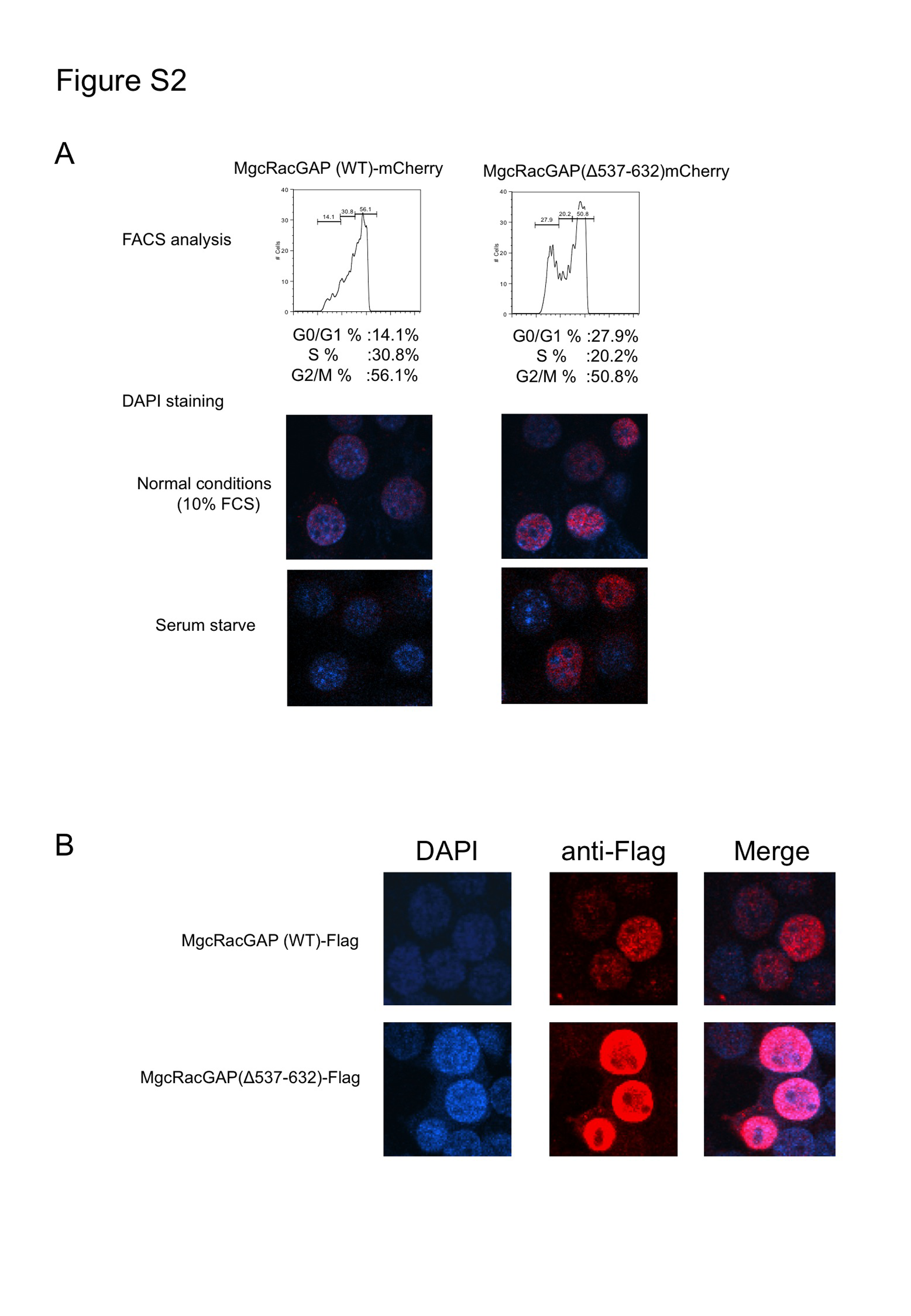

Supplement: Figure S2 — MgcRacGAP (Δ537–632)-mCherry is not degraded in the G0/G1 phase and localized to the nucleus. (A) NIH3T3 cells transduced with pMXs-IRES-Puror-MgcRacGAP (WT)-mCherry or MgcRacGAP (Δ537–632)-mCherry were stained with Hoechest 33342 and analyzed with FACS (top panel). DAPI staining was subjected to microscopic analysis by Olympus IX71 and Fluoview with X 100 Objective lens (Olympus, Tokyo, Japan) (middle: normal conditions, bottom: serum starvation). (B) NIH3T3 cells transduced with pMXs-IRES-Puror-MgcRacGAP (WT)-Flag or MgcRacGAP (Δ537–632)-Flag were stained with DAPI and anti-Flag (M2) and view with Olympus IX71 and Fluoview with X 100 Objective lens. (TIF) [file pone.0063001.s002.tif]
