# Supplementary material for: APCCDH1 Targets MgcRacGAP for Destruction in the Late M Phase
Source: PLoS One. 2013 May 16;8(5):e63001. doi: 10.1371/journal.pone.0063001 (PMC3656054; doi:10.1371/journal.pone.0063001)
Supplement: Protocol S1 — Hoechst staining and cell cycle analysis. NIH3T3 cells transduced with MgcRacGAP (WT)-mCherry or MgcRacGAP (Δ537–632)-mCherry were stained with 5 µg/ml Hoechst 33342 (Invitrogen, Carlsbad, CA) and 20 µg/ml verapamil (Sigma-Aldrich, St. Louis, USA). After incubation for 30 min, DNA content of mCherry (+) cells was analyzed by FACSAria (BD Biosciences, San Jose, USA). (DOC) [file pone.0063001.s006.doc]

**Protocol S1.**

Hoechst staining and cell cycle analysis.

NIH3T3 cells transduced with MgcRacGAP (WT)-mCherry or MgcRacGAP (Δ537-632)-mCherry were stained with 5 μg/ml Hoechst 33342 (Invitrogen, Carlsbad, CA) and 20 μg/ml verapamil (Sigma-Aldrich, St. Louis, USA). After incubation for 30 min, DNA content of mCherry (+) cells was analyzed by FACSAria (BD Biosciences, San Jose, USA).

Immunocytochemistry

Immunocytochemistry were performed as described previously [13].
